# Sex Differences in Alzheimer’s Disease Risk Factors in the Southern Community Cohort Study

**DOI:** 10.1093/geroni/igaf122.4278

**Published:** 2025-12-31

**Authors:** Kelsie Full, Xijing Han, Hui Shi, Danxia Yu, Logan Dumitrescu, Atul Malhotra, Loren Lipworth

**Affiliations:** Vanderbilt University Medical Center, Nashville, Tennessee, United States; Vanderbilt University Medical, Nashville, Tennessee, United States; Vanderbilt University Medical, Nashville, Tennessee, United States; Vanderbilt University Medical, Nashville, Tennessee, United States; Vanderbilt University Medical, Nashville, Tennessee, United States; University of California, San Diego, School of Medicine, La Jolla, Florida, United States; Vanderbilt University Medical, Nashville, Tennessee, United States

## Abstract

In the United States, aging women bear a disproportionate Alzheimer’s disease (AD) and related dementias (ADRD) burden, accounting for nearly 2/3 of cases. This striking sex disparity remains poorly understood and may be driven by differences in ADRD risk factors. In the Southern Community Cohort Study (SCCS), we investigated the sex-stratified prevalence of the 2024 Lancet Commission on Dementia identified ADRD risk factors and associations with incident ADRD. This analysis includes 28,250 SCCS participants (mean age: 60 years; 62% females; 59% Black) who reached ≥65 years prior to end of follow-up and were continuously enrolled in Medicare. ADRD risk factors included education, low physical activity, high sedentary time, smoking, alcohol use, obesity, social isolation, sleep duration, and physician diagnosis of depression, hypertension, and hypocholesterolemia. Incident ADRD was ascertained via Centers for Medicare and Medicaid Services linkage through December 2020. We ran Cox proportional hazard models adjusting for age, race, education, employment and income. Compared to males, females were significantly more likely to have depression, hypercholesterolemia, diabetes, hypertension, obesity, social isolation and short sleep duration (all p-values < 0.001). Over 4.5 years of follow-up, 1,994 ADRD cases occurred among females and 1,095 cases among males. Associations with incident ADRD were stronger among females compared to males for higher education (Hazard Ratios (HRs): 1.33; 1.19), hypercholesterolemia (HRs:1.10; 0.98), depression (HRs: 1.60; 1.39), higher sedentary time (HRs: 1.31; 1.18), hypertension (HRs:1.24; 1.12), and social isolation (HRs: 1.25; 1.19). ADRD risk factors that are more prevalent and impactful in females may inform novel prevention strategies.

